# On Global Attraction to Stationary States for Wave Equations with Concentrated Nonlinearities

**DOI:** 10.1007/s10884-016-9563-1

**Published:** 2016-11-21

**Authors:** Elena Kopylova

**Affiliations:** 1 Faculty of Mathematics, Vienna University, Vienna, Austria; 2 Institute for Information Transmission Problems, Russian Academy of Sciences, Moscow, Russia

**Keywords:** Wave equation, Point interaction, Stationary states, Global attraction, 35B25, 35B41, 35L70

## Abstract

The global *attraction* to stationary states is established for solutions to 3D wave equations with concentrated nonlinearities: each finite energy solution converges as $t\rightarrow \pm \infty$ to stationary states. The attraction is caused by nonlinear energy radiation.

## Introduction

The paper concerns a nonlinear interaction of the *real wave field* with a point oscillator.The system is governed by the following equations

1.1 $$\begin{aligned} \left\{ \begin{array}{c} \ddot{\psi }(x,t)=\Delta \psi (x,t)+\zeta (t)\delta (x)\\ \lim \limits _{x\rightarrow 0}(\psi (x,t)-\zeta (t)G(x))=F(\zeta (t)) \end{array}\right| \quad x\in {\mathbb R}^3,\quad t\in {\mathbb R}, \end{aligned}$$

where *G* is the Green’s function of operator $-\Delta$ in ${\mathbb R}^3$, i.e.

$$\begin{aligned} G(x)=\frac{1}{4\pi |x|}, \end{aligned}$$

All derivatives here and below are understood in the sense of distributions. The nonlinearity admits a potential

1.2 $$\begin{aligned} F(\zeta )=U'(\zeta ), \quad \zeta \in {\mathbb R},\quad U\in C^2({\mathbb R}). \end{aligned}$$

We assume that

1.3 $$\begin{aligned} U(\zeta )\rightarrow \infty ,\quad \zeta \rightarrow \pm \infty . \end{aligned}$$

Furthermore, we assume that the set $Q=\{q\in R: F(q)=0\}$ is nonempty. Then the system (1.1) admits stationary solutions *qG*(*x*), where $q\in Q$. We suppose that the set *Q* satisfies the following condition

1.4 $$\begin{aligned}{}[a,b]\not \subset Q~\mathrm{for}~ \mathrm{any}~~ a<b. \end{aligned}$$

Let  be the completion of the space $C_0^\infty ({\mathbb R}^3)$ in the norm $\Vert \nabla \psi (x)\Vert _{L^2({\mathbb R}^3)}$. Equivalently, using Sobolev’s embedding theorem, , and

1.5 $$\begin{aligned} \Vert f\Vert _{L^6({\mathbb R}^3)}\le C\Vert \nabla f\Vert _{L^2({\mathbb R}^3)}. \end{aligned}$$

DenoteWe consider Cauchy problem for system (1.1) with initial data $\Psi (x,0)=(\psi (x,0),{\dot{\psi }}(x,0))$ which can be represented as the sum of *regular* component from  and *singular* component proportional to *G*(*x*) (see Definition 2.1). Our main goal is the global attraction of the solution $\Psi (x,t)=(\psi (x,t),{\dot{\psi }}(x,t))$ to stationary states:

$$\begin{aligned} \Psi (x,t)\rightarrow (q_{\pm }G(x),\,0),\quad t\rightarrow \pm \infty ,\quad q_{\pm }\in Q, \end{aligned}$$

where the asymptotics hold in local $L^2\oplus L^2$-seminorms.

Similar global attraction was established for the first time (i) in [6–8] for 1D wave and Klein–Gordon equations coupled to nonlinear oscillators, (ii) in [9, 10] for nD Klein-Gordon and Dirac equations with mean field interaction, and (iii) in [5] for discrete in space and time nD Klein–Gordon equation equations interacting with a nonlinear oscillator.

In the context of the Schrödinger and wave equations the point interaction of type (1.1) was introduced in [1, 2, 4, 11, 12], where the well-posedness of the Cauchy problem and the blow up solutions were studied. The orbital and asymptotic stability of soliton solutions for the Schrödinger equation with the point interaction has been established in [3]. The global attraction for 3D equations with the point interaction was not studied up to now. In the present paper we prove for the first time the global attraction in the case of 3D wave equation.

Let us comment on our approach. First, similarly to [8–10], we represent the solution as the sum of *dispersive* and *singular* components. The dispersive component is a solution of the free wave equation with the same initial data $\Psi (x,0)$. The singular component is a solution of a coupled system of wave equation with zero initial data and a point source, and of a nonlinear ODE.

We prove the long-time decay of the dispersive component in local $H^2\oplus H^1$-seminorms. To establish the decay for regular part of the dispersive component, corresponding to regular initial data from $H^2\oplus H^1$, we apply the strong Huygens principle and the energy conservation for the free wave equation. For the remaining singular part we apply the strong Huygens principle. The dispersive decay is caused by the energy radiation to infinity.

Finally, we study the nonlinear ODE with a source. We prove that the source decays and then the attractor of the ODE coincides with the set of zeros of the nonlinear function *F*, i.e. with the set *Q*. This allows us to prove the convergence of the singular component of the solution to one of the stationary solution in local $L^2\oplus L^2$-seminorms.

## Main Results

### Model

We fix a nonlinear function $F:{\mathbb R}\rightarrow {\mathbb R}$ and define the domain2.1which generally is not a linear space. The limit in (2.1) is well defined since  by the Sobolev embedding theorem.

Let $H_F$ be a nonlinear operator on the domain $D_F$ defined by

2.2 $$\begin{aligned} H_F \psi =\Delta \psi _{reg},\quad \psi \in D_F. \end{aligned}$$

The system (1.1) for $\psi (t)\in D_F$ reads

2.3 $$\begin{aligned} \ddot{\psi }(x,t)=H_F \psi (x,t),\quad x\in {\mathbb R}^3,\quad t\in {\mathbb R}. \end{aligned}$$

Let us introduce the phase space for Eq. (2.3). Denote the spaceObviously, $D_F\subset {\dot{D}}$.

#### Definition 2.1

${\mathscr {D}}_F$ is the Hilbert space of the states $\Psi =(\psi (x),\pi (x))\in D_F\oplus {\dot{D}}$ equipped with the finite norm

$$\begin{aligned} \Vert \Psi \Vert _\mathcal{D}^2{:\,=}\,\Vert \nabla \psi _{reg} \Vert _{L^2({\mathbb R}^3)}^2+\Vert \Delta \psi _{reg} \Vert _{L^2({\mathbb R}^3)}^2 +\Vert \nabla \pi _{reg} \Vert _{L^2({\mathbb R}^3)}^2+|\zeta |^2+|\eta |^2. \end{aligned}$$

### Well-Posedness

#### Theorem 2.2

Let conditions (1.2) and (1.3) hold. Then

(i) For every initial data $\Psi (0)=(\psi (0),{\dot{\psi }}(0))\in {\mathscr {D}}_F$ the Eq. (2.3) has a unique strong solution $\psi (t)$ such that
(ii) The energy is conserved:
  $$\begin{aligned} {\mathscr {H}}_F(\Psi (t)){:\,=} \,\frac{1}{2} \Big (\Vert {\dot{\psi }}(t)\Vert ^2_{L^2({\mathbb R}^3)} +\Vert \nabla \psi _{reg}(t)\Vert ^2_{L^2({\mathbb R}^3)}\Big )+U(\zeta (t))=\mathrm{const}, \quad t\in {\mathbb R}. \end{aligned}$$
(iii) The following a priori bound holds
  2.4 $$\begin{aligned} |\zeta (t)|\le C(\Psi (0)), \quad t\in R. \end{aligned}$$

This result is proved in [12, Theorem 3.1]. For the convenience of readers, we sketch main steps of the proof in Appendix in the case $t\ge 0$ clarifying some details of [12]. As the result the solution $\psi (x,t)$ to (2.3) with initial data $\psi (0)=\psi _0\in D_F$, ${\dot{\psi }}(0)=\pi _0\in {\dot{D}}$ can be represented as the sum

2.5 $$\begin{aligned} \psi (x,t){:\,=} \,\psi _f(x,t)+\psi _S(x,t), \quad t\ge 0, \end{aligned}$$

where the *dispersive component* $\psi _f(x,t)$ is a unique solution of the Cauchy problem for the free wave equation

2.6 $$\begin{aligned} \ddot{\psi }_f(x,t) = \Delta \psi _f(x,t), \quad \psi _f(x,0) = \psi _0(x),\quad {\dot{\psi }}_f(x,0) = \pi _0(x), \end{aligned}$$

and the *singular component* $\psi _S(x,t)$ is a unique solution of the Cauchy problem for the wave equation with a point source

2.7 $$\begin{aligned} \ddot{\psi }_S(x,t)= \Delta \psi _S(x,t) +\zeta (t)\delta (x), \quad \psi _S(x,0) = 0,\quad {\dot{\psi }}_S(x,0)=0. \end{aligned}$$

Here $\zeta (t)\in C^1_b([0,\infty ))$ is a unique solution to the Cauchy problem for the following first-order nonlinear ODE

2.8 $$\begin{aligned} \frac{1}{4\pi }{\dot{\zeta }}(t)+ F(\zeta (t))=\lambda (t), \quad \zeta (0)=\zeta _0, \end{aligned}$$

where

2.9 $$\begin{aligned} \lambda (t){:\,=}\lim \limits _{x\rightarrow 0}\psi _f(x,t),\quad t>0, \end{aligned}$$

Next lemma implies that limit (2.9) is well defined, and there exists $\lambda (0+)=\lim \limits _{t\rightarrow 0+}\lambda (t)$.

#### Lemma 2.3

Let $$(\psi _0,\pi _0)\in {\mathscr {D}}_F$$. Then

(i) There exists a unique solution $\psi _f\in C([0;\infty ), L^2_{loc})$ to (2.6).
(ii) The limit in (2.9) exists and is continuous in $t\in [0,\infty )$.
(iii) $${\dot{\lambda }}\in L^2_{loc}([0,\infty ))$$.

#### Proof

(i) We split $\psi _f(x,t)$ as

$$\begin{aligned} \psi _f(x,t)=\psi _{f,reg}(x,t)+g(x,t), \end{aligned}$$

where $\psi _{f,reg}$ and *g* are the solutions to the free wave equation with initial data  and $(\zeta _0 G,{\dot{\zeta }}_0 G)$, respectively. By the energy conservation . Now we obtain an explicit formula for *g*(*x*, *t*). Note that $h(x,t)=g(x,t)-\xi (t)G(x)$, where $\xi (t)=\zeta _0+t{\dot{\zeta }}_0$, satisfies

2.10 $$\begin{aligned} \ddot{h}(x,t)=\Delta h(x,t)-\xi (t)\delta (x) \end{aligned}$$

with zero initial data. The unique solution to (2.10) is the spherical wave

2.11 $$\begin{aligned} h(x,t)=-\frac{\theta (t-|x|)}{4\pi |x|}\xi (t-|x|),\quad t\ge 0, \end{aligned}$$

where $\theta$ is the Heaviside function. This is well-known formula [14, Section 175] for the retarded potential of the point particle. Hence,

$$\begin{aligned} g(x,t)= & {} h(x,t)+\xi (t)G(x)=-\frac{\theta (t-|x|)(\zeta _0+(t-|x|){\dot{\zeta }}_0)}{4\pi |x|}\nonumber \\&\quad +\frac{\zeta _0+t{\dot{\zeta }}_0}{4\pi |x|}\in C([0,\infty ),L^2_{loc}({\mathbb R}^3)). \end{aligned}$$

(ii) We have

2.12 $$\begin{aligned} \lim \limits _{x\rightarrow 0}g(x,t)={\dot{\zeta }}_0/(4\pi ),\quad t>0. \end{aligned}$$

Moreover, for any $t\ge 0$ the $\lim \limits _{x\rightarrow 0}\psi _{f,reg}(x,t)$ exists because .

(iii) Due to (2.12) it remains to show that ${\dot{\psi }}_{f,reg}(0,t)\in L^2_{loc}([0,\infty ))$. This follows immediately from [12, Lemma 3.4]. $\square$

### Stationary Solutions and the Main Theorem

The stationary solutions of Eq. (2.3) are solutions of the form

2.13 $$\begin{aligned} \psi _q(x)=qG(x)\in L^2_{loc}({\mathbb R}^3),\quad q\in {\mathbb R}. \end{aligned}$$

#### Lemma 2.4

(Existence of stationary solutions). Function (2.13) is a stationary soliton to (2.3) if and only if

2.14 $$\begin{aligned} F(q)=0. \end{aligned}$$

#### Proof

Evidently, $\psi _q(x)$ admits the splitting $\psi _q(x)=\psi _{reg}(x,t)+\zeta (t)G(x)$, $\psi _{reg}(x,t)\equiv 0$ and $\zeta (t)\equiv q$. Hence, the second equation of (1.1) is equivalent to (2.14). $\square$

Our main result is the following theorem.

#### Theorem 2.5

(Main Theorem) Let assumptions (1.2), (1.3) and (1.4) hold and let $\psi (x,t)$ be a solution to eq. (2.3) with initial data $\Psi (0)=(\psi (0),\,{\dot{\psi }}(0))\in {\mathscr {D}}_F$. Then

$$\begin{aligned} (\psi (t),\,{\dot{\psi }}(t))\rightarrow (\psi _{q_{\pm }},\,0),\quad t\rightarrow \pm \infty ,\quad q_{\pm }\in Q, \end{aligned}$$

where the convergence hold in $L^2_{loc}({\mathbb R}^3)\oplus L^2_{loc}({\mathbb R}^3)$.

It suffices to prove Theorem 2.5 for $t\rightarrow +\infty$.

## Dispersion Component

We will only consider the solution $\psi (x,t)$ restricted to $t\ge 0$. In this section we extract regular and singular parts from the dispersion component $\psi _f(x,t)$ and establish their local decay. First, we represent the initial data $(\psi (0),\,{\dot{\psi }}(0))=(\psi _0,\pi _0)\in {\mathscr {D}}_F$ as

$$\begin{aligned} (\psi _0,~\pi _0)=(\psi _{0,reg},~\pi _{0,reg})+(\zeta _0G, ~{\dot{\zeta }}_0 G) =(\varphi _{0},~\eta _{0})+(\zeta _0\chi G, ~{\dot{\zeta }}_0 \chi G), \end{aligned}$$

where a cut-of function $\chi \in C_0^\infty ({\mathbb R}^3)$ satisfies

3.1 $$\begin{aligned} \chi (x)=\left\{ \begin{array}{ll} 1,\quad |x|\le 1\\ 0,\quad |x|\ge 2 \end{array}\right. \end{aligned}$$

Let us show that

3.2 $$\begin{aligned} (\varphi _{0},~\eta _{0})\in H^2({\mathbb R}^3)\oplus H^1({\mathbb R}^3). \end{aligned}$$

Indeed,

$$\begin{aligned} (\varphi _{0},~\eta _{0})=(\psi _0-\zeta _0\chi G,~\pi _0-{\dot{\zeta }}_0\chi G) \in L^2({\mathbb R}^3)\oplus L^2({\mathbb R}^3), \end{aligned}$$

On the other hand,Now we split the dispersion component $\psi _f(x,t)$ as

3.3 $$\begin{aligned} \psi _f(x,t)=\varphi (x,t)+\psi _{G}(x,t), \quad t\ge 0, \end{aligned}$$

where $\varphi$ and $\psi _{G}$ are defined as solutions to the following Cauchy problems:

3.4 $$\begin{aligned}&\ddot{\varphi }(x,t)=\Delta \varphi (x,t), \qquad (\varphi ,{\dot{\varphi }})\vert _{_{t=0}}=(\varphi _{0},~\eta _{0}), \end{aligned}$$

3.5 $$\begin{aligned}&\ddot{\psi }_{G}(x,t)=\Delta \psi _{G}(x,t), \qquad (\psi _{G},{\dot{\psi }}_{G})\vert _{_{t=0}}=(\zeta _0\chi G, {\dot{\zeta }}_0\chi G), \end{aligned}$$

and study the decay properties of $\psi _{G}$ and $\varphi$.

### Lemma 3.1

For the solution $\psi _{G}(x,t)$ to (3.5) the strong Huygens principle holds:

3.6 $$\begin{aligned} \psi _{G}(x,t)=0 ~~\mathrm{for}~~t\ge |x|+2. \end{aligned}$$

### Proof

The solution $\varphi _G(x,t)$ to the free wave equation with initial data $(0, \chi G)\in H^1({\mathbb R}^3)\oplus L^2({\mathbb R}^3)$ satisfies the strong Huygens principle due to [13, Theorem XI.87]. Further,

$$\begin{aligned} \psi _G(x,t)=\zeta _0{\dot{\varphi }}_G(x,t)+{\dot{\zeta }}_0\varphi _G(x,t). \end{aligned}$$

Then (3.6) follows. $$\square $$

The following lemma states a local decay of solutions to the free wave equation with regular initial data from $H^2({\mathbb R}^3)\oplus H^1({\mathbb R}^3)$.

### Lemma 3.2

Let $\varphi (t)$ be a solution to (3.4) with initial data $\phi _0=(\varphi _0,\eta _0)\in H^2({\mathbb R}^3)\oplus H^1({\mathbb R}^3)$. Then

3.7 $$\begin{aligned} \left\| (\varphi (t),{\dot{\varphi }}(t)) \right\| _{H^2(B_R)\oplus H^1(B_R)}\rightarrow 0,\quad t\rightarrow \infty ,\quad \forall R>0, \end{aligned}$$

where $B_R$ is the ball of radius *R*.

### Proof

For any $r\ge 1$ denote $\chi _r=\chi (x/r)$, where $\chi (x)$ is a cut-off function defined in (3.1). Let $u_r(t)$ and $v_r(t)$ be the solutions to the free wave equations with the initial data $\chi _r \phi _0$ and $(1-\chi _r) \phi _0$, respectively, so that $u(t)=u_r(t)+v_r(t)$. By the strong Huygens principle

$$\begin{aligned} u_r(x,t)=0 ~~\mathrm{for}~~t\ge |x|+2r. \end{aligned}$$

To conclude (3.7), it remains to note that3.8due to the energy conservation for the free wave equation. We also use the embedding . The right-hand side of (3.8) could be made arbitrarily small if $r\ge 1$ is sufficiently large. $\square$

Finally, (3.3) , (3.6) , (3.2) and Lemma 3.2 imply

3.9 $$\begin{aligned} \left\| (\psi _f(t),{\dot{\psi }}_f(t)) \right\| _{H^2(B_R)\oplus H^1(B_R)}\rightarrow 0,\quad t\rightarrow \infty ,\quad \forall R>0. \end{aligned}$$

## Singular Component

Due to (3.9) to prove Theorem 2.5 it suffices to deduce the convergence to stationary states for the singular component $\psi _S(x,t)$ of the solution.

### Proposition 4.1

Let assumptions of Theorem 2.5 hold, and let $\psi _S(t)$ be a solution to (2.7). Then

$$\begin{aligned} (\psi _S(t),{\dot{\psi }}_S(t))\rightarrow (\psi _{q_{\pm }},~0),\quad t\rightarrow \infty , \end{aligned}$$

where the convergence holds in $L^2_{loc}({\mathbb R}^3)\oplus L^2_{loc}({\mathbb R}^3)$.

### Proof

The unique solution to (2.7) is the spherical wave

4.1 $$\begin{aligned} \psi _S(x,t)=\frac{\theta (t-|x|)}{4\pi |x|}\zeta (t-|x|),\quad t\ge 0, \end{aligned}$$

cf. (2.10–2.11). Then a priori bound (2.4) and Eq. (2.8) imply that

$$\begin{aligned} (\psi _S(t),{\dot{\psi }}_S(t))\in L^2(B_R)\oplus L^2(B_R),\quad 0\le R<t. \end{aligned}$$

First, we obtain a convergence of $\zeta (t)$.$\square$

### Lemma 4.2

There exists the limit

4.2 $$\begin{aligned} \zeta (t)\rightarrow q_+,\quad t\rightarrow \infty , \end{aligned}$$

where $$q_+\in Q$$.

### Proof

From (2.4) it follows that $\zeta (t)$ has the upper and lower limits:

$$\begin{aligned} \underline{\lim }_{t\rightarrow \infty }\zeta (t)=a,\quad \overline{\lim }_{t\rightarrow \infty }\zeta (t)=b. \end{aligned}$$

Suppose that $a<b$. Then the trajectory $\zeta (t)$ oscillates between *a* and *b*. Assumption (1.4) implies that $F(\zeta _0)\not =0$ for some $\zeta _0\in (a,b)$. For the concreteness, let us assume that $F(\zeta _{0})>0$. The convergence (3.9) implies that

4.3 $$\begin{aligned} \lambda (t)=\psi _f(0,t)\rightarrow 0,\qquad t\rightarrow \infty . \end{aligned}$$

Hence, for sufficiently large *T* we have

$$\begin{aligned} -F(\zeta _{0})+\lambda (t)<0, \quad t\ge T. \end{aligned}$$

Then for $t\ge T$ the transition of the trajectory from left to right through the point $\zeta _0$ is impossible by (2.8). Therefore, $a=b=q_+$. Finally $F(q_+)=0$ by (2.8). $\square$

Further,

4.4 $$\begin{aligned} \theta (t-|x|)\rightarrow 1, \quad t\rightarrow \infty \end{aligned}$$

uniformly in $|x|\le R$. Then (4.1) and (4.2) imply that

$$\begin{aligned} \psi _S(t)\rightarrow q_+G, \quad t\rightarrow \infty , \end{aligned}$$

where the convergence holds in $L^2_{loc}({\mathbb R}^3)$. It remains to deduce the convergence of ${\dot{\psi }}_S(t)$. We have

$$\begin{aligned} {\dot{\psi }}_S(x,t)=\frac{\theta (t-|x|)}{4\pi |x|}{\dot{\zeta }}(t-|x|), \quad t>|x|. \end{aligned}$$

From (4.2), (2.8) and (4.3) it follows that ${\dot{\zeta }}(t)\rightarrow 0$ as $t\rightarrow \infty$. Then

$$\begin{aligned} {\dot{\psi }}_S(t)\rightarrow 0,\qquad t\rightarrow \infty \end{aligned}$$

in $L^2_{loc}({\mathbb R}^3)$ by (4.4). This completes the proof of Proposition 4.1 and Theorem 2.5. $\square$

## Appendix

Here we sketch main steps of the proof [12, Theorem 3.1]. First we adjust the nonlinearity *F* so that it becomes Lipschitz-continuous. Define

5.1 $$\begin{aligned} \Lambda (\Psi _0)=\sup \{|\zeta |: \zeta \in {\mathbb R},\, U(\zeta )\le H_F(\Psi _0)\},\end{aligned}$$

where $\Psi _0=\Psi (0)\in {\mathscr {D}}_F$ is the initial data from Theorem 2.2. Then we may pick a modified potential function ${\tilde{U}}(\zeta )\in C^2({\mathbb R})$, so that

5.2 $$\begin{aligned} \left\{ \begin{array}{lll} &{}\tilde{U}(\zeta )= U(\zeta ),\quad &{}|\zeta |\le \Lambda (\Psi _0)\\ &{}\tilde{U}(\zeta )>H_F(\Psi _0),\quad &{}|\zeta |>\Lambda (\Psi _0), \end{array}\right. \end{aligned}$$

and the function ${\tilde{F}}(\zeta )={\tilde{U}}'(\zeta )$ is Lipschitz continuous:

5.3 $$\begin{aligned} |{\tilde{F}}(\zeta _1)-{\tilde{F}}(\zeta _2)|\le C|\zeta _1-\zeta _2|,\quad \zeta _1,\zeta _2\in {\mathbb R}. \end{aligned}$$

We consider the Cauchy problem for (2.3)) with the modified nonlinearity ${\tilde{F}}$. According to Lemma 2.3 there exist the unique solution $\psi _f(x,t)\in C([0,\infty ),L^2_{loc}({\mathbb R}^3))$ to (2.6) and $\lambda (t)=\lim \limits _{x\rightarrow 0}\psi _f(x,t)\in C([0,\infty ))$. The following lemma follows by the contraction mapping principle.

### Lemma 5.1

Let conditions (5.2–5.3) be satisfies. Then there exists $\tau >0$ such that the Cauchy problem

5.4 $$\begin{aligned} \frac{1}{4\pi }{\dot{\zeta }}(t)+{\tilde{F}}(\zeta (t))=\lambda (t),\quad \zeta (0)=\zeta _{0} \end{aligned}$$

has a unique solution $\zeta \in C^1([0,\tau ])$.

Denote

$$\begin{aligned} \psi _S(t,x){:\,=}\frac{\theta (t-|x|)}{4\pi |x|}\zeta (t-|x|), \quad t\in [0,\tau ], \end{aligned}$$

with $\zeta$ from Lemma 5.1. Now we establish the local well-posedness.

### Proposition 5.2

Let the conditions (5.2)–(5.3) hold. Then the function $\psi (x,t)\,{:=}\, \psi _f(x,t)+\psi _S(x,t)$ is a unique strong solution to the system

5.5 $$\begin{aligned} \left\{ \begin{array}{c} \ddot{\psi }(x,t)=\Delta \psi (x,t)+\zeta (t)\delta (x)\\ \\ \lim \limits _{x\rightarrow 0}(\psi (x,t)-\zeta (t)G(x))={\tilde{F}}(\zeta (t)) \end{array}\right| \quad x\in {\mathbb R}^3,\quad t\in [0,\tau ]. \end{aligned}$$

with initial data

$$\begin{aligned} \psi (0) = \psi _0\in D_{{\tilde{F}}},\quad {\dot{\psi }}(0) = \pi _0\in {\dot{D}}, \end{aligned}$$

and satisfies

5.6 $$\begin{aligned} (\psi (t),{\dot{\psi }}(t))\in {\mathscr {D}}_{{\tilde{F}}},\quad t\in [0,\tau ]. \end{aligned}$$

### Proof

Since $\zeta (t)$ solves (5.4) one has

5.7 $$\begin{aligned} \lim _{x \rightarrow 0}\, ( \psi (t,x)\,- zeta(t) G(x))= & {} \lambda (t)+ \lim _{x\rightarrow 0}\Big (\frac{\theta (t-|x|)\zeta (t-|x|)}{4\pi |x|}-\frac{\zeta (t)}{4\pi |x|}\Big )\nonumber \\= & {} \lambda (t)-\frac{1}{4\pi }{\dot{\zeta }}(t)=\tilde{F}(\zeta (t)). \end{aligned}$$

Therefore, the second equation of (5.5) is satisfied. Further,

$$\begin{aligned} \ddot{\psi }=\ddot{\psi }_f+\ddot{\psi }_S=\Delta \psi _f +\Delta \psi _S+\zeta \delta =\Delta \psi +\zeta \delta \end{aligned}$$

and $\psi$ solves the first equation of (5.5) then. Let us check (5.6). Note that the function $\psi _{reg,1}(x,t)=\psi (x,t)-\zeta (t) G_1(x)$, where $G_1(x)=G(x)e^{-|x|}$, is a solution to

$$\begin{aligned} \ddot{\psi }_{reg,1}(x,t)=\Delta \psi _{reg,1}(x,t)+(\zeta (t)-\ddot{\zeta }(t)) G_1(x) \end{aligned}$$

with initial data from $H^2\oplus H^1$. Lemma 2.3-(iii) and Eq. (5.4) imply that $\ddot{\zeta }\in L^2([0,\tau ])$. Hence,

$$\begin{aligned} (\psi _{reg,1}(x,t),{\dot{\psi }}_{reg,1}(x,t))\in H^2\oplus H^1,\quad t\in [0,\tau ] \end{aligned}$$

by [12, Lemma 3.2]. Therefore,

$$\begin{aligned} \psi _{reg}(x,t)=\psi (x,t)-\zeta (t) G(x)=\psi _{reg,1}(x,t)+\zeta (t)(G_1(x)-G(x)) \end{aligned}$$

satisfies , and (5.6) holds then.

Suppose now that $\tilde{\psi }=\tilde{\psi }_{reg}+\tilde{\zeta } G$, such that $(\tilde{\psi },{\dot{\tilde{\psi }})}\in {\mathscr {{D}}}_{\tilde{F}}$, is another strong solution of (5.5). Then, by reversing the above argument, the second equation of (5.5) implies that $\tilde{\zeta }$ solves the Cauchy problem (5.4). The uniqueness of the solution of (5.4) implies that $\tilde{\zeta }=\zeta$. Then, defining

$$\begin{aligned} \psi _S(t,x)\,{:=}\,\frac{\theta (t-|x|)}{4\pi |x|}\zeta (t-|x|), \quad t\in [0,\tau ], \end{aligned}$$

for $\tilde{\psi }_f=\tilde{\psi }-\psi _S$ one obtains

$$\begin{aligned} \ddot{\tilde{\psi }}_f=\ddot{\tilde{\psi }}-\ddot{\psi }_S=\Delta \tilde{\psi }_{reg}-(\Delta \psi _S+\zeta \delta )= \Delta (\tilde{\psi }_{reg}-(\psi _S-\zeta {G}))=\Delta \tilde{\psi }_f\,, \end{aligned}$$

i.e $\tilde{\psi }_f$ solves the Cauchy problem (2.6). Hence, $\tilde{\psi }_f=\psi _f$ by the uniqueness of the solution to (2.6), and then $\tilde{\psi }=\psi$. $\square$

According to [12, Lemma 3.7]

5.8 $$\begin{aligned} {\mathscr {H}}_{\tilde{F}}(\Psi (t))=\Vert {\dot{\psi }}(t)\Vert ^2+\Vert \nabla \psi _{reg}(t)\Vert ^2 +\tilde{U}(\zeta (t))=const,\quad t\in [0,\tau ]. \end{aligned}$$

### Lemma 5.3

The following identity holds

5.9 $$\begin{aligned} {\tilde{U}}(\zeta (t))=U(\zeta (t)), \quad t\in [0,\tau ]. \end{aligned}$$

### Proof

First note that

$$\begin{aligned} {\mathscr {H}}_{F}(\Psi _0)\ge U(\zeta _{0}). \end{aligned}$$

Therefore, $|\zeta _0|\le \Lambda (\Psi _0)$, and then ${\tilde{U}}(\zeta _0)=U(\zeta _0)$, ${\mathscr {H}}_{{\tilde{F}}}(\Psi _0)={\mathscr {H}}_{F}(\Psi _0)$. Further,

$$\begin{aligned} {\mathscr {H}}_{{F}}(\Psi _0)={\mathscr {H}}_{\tilde{F}}(\Psi (t))\ge {\tilde{U}}(\zeta (t)),\quad t\in [0,\tau ]. \end{aligned}$$

Hence (5.8) implies that

5.10 $$\begin{aligned} |\zeta (t)|\le \Lambda (\Psi _0), \quad t\in [0,\tau ]. \end{aligned}$$

$$\square $$

From the identity (5.9) it follows that we can replace ${\tilde{F}}$ by *F* in Proposition 5.2 and in (5.8). The solution $\Psi (t)=(\psi (t),{\dot{\psi }}(t))\in {\mathscr {D}}$ constructed in Proposition 5.2 exists for $0\le t\le \tau$, where the time span $\tau$ in Lemma 5.1 depends only on $\Lambda (\Psi _0)$. Hence, the bound (5.10) at $t=\tau$ allows us to extend the solution $\Psi$ to the time interval $[\tau , 2\tau ]$. We proceed by induction to obtain the solution for all $t\ge 0$.
